# A Proinflammatory Diet Is Associated with Higher Risk of Peripheral Artery Disease

**DOI:** 10.3390/nu14173490

**Published:** 2022-08-25

**Authors:** Heze Fan, Juan Zhou, Yuzhi Huang, Xueying Feng, Peizhu Dang, Guoliang Li, Zuyi Yuan

**Affiliations:** 1 Cardiovascular Department, First Affiliated Hospital of Xi’an Jiao Tong University, Xi’an 710061, China; 2 Key Laboratory of Environment and Genes Related to Diseases, Ministry of Education, Xi’an 710061, China

**Keywords:** dietary inflammatory index, peripheral arterial disease, National Health and Nutrition Examination Survey, cross-sectional study, relationship

## Abstract

Peripheral arterial disease (PAD) has a strong relationship with inflammation. However, it is unclear whether the dietary inflammatory potential is associated with PAD. We aimed to address this knowledge gap. The dietary inflammatory index (DII) was obtained using a 24-h dietary recall interview for each individual. Logistic regression models and restricted cubic spline were performed to assess the relationship of DII with the prevalence of PAD. In addition, Spearman correlation analysis and subgroup analysis were also undertaken. In total, 5840 individuals from the 1999–2004 National Health and Nutrition Examination Survey (NHANES) were enrolled in our study. Participants in higher DII quartile tended to have higher rates of PAD. The increase in DII scores showed a positive association with PAD after fully multivariate adjustment (OR (odds ratios) = 1.094, 95% confidence interval (CI): 1.022–1.171). The multivariable-adjusted OR and 95% CI of the highest DII index quartile compared with the lowest quartile was 1.543 (95% CI: 1.116–2.133). Subgroup analysis demonstrated that the positive association between DII and PAD was persistent across population subgroups. In conclusion, we report that a proinflammatory dietary pattern is related to a higher risk of developing PAD among US adults.

## 1. Introduction

Peripheral arterial disease (PAD) is a type of atherosclerosis characterized by fatty deposits along the walls of arteries, leading to narrowing and obstructive lesions in the lumen, primarily damaging the arteries of the lower extremities and feet [1]. In 2018, Jun Shu et al. estimated that more than 200 million people worldwide suffer from PAD [2]. PAD not only causes lower limb ischemic ulcers and amputations but also significantly increases the risk of cardiovascular and cerebrovascular events and death in patients [3,4]. The economic burden and health hazards caused by PAD cannot be ignored. Therefore, it is crucial to determine the etiology of PAD and take adequate measures. Previous studies have shown that inflammatory markers, endothelial dysfunction, and oxidative stress play an important role in the development of PAD [5,6,7,8,9,10,11]. Meanwhile, it was reported that dietary pattern, evaluated by the dietary inflammatory index (DII), was associated with various inflammatory markers, including CRP, IL-6, and homocysteine [12,13]. In addition, higher DII scores have been shown to be associated with poor health and higher risk of diseases, such as obesity [14], various cancer [15,16], cardiovascular disease (CVD) [14,17], chronic obstructive pulmonary disease [18], depression [19,20], metabolic syndrome [21], type 2 diabetes [14,22], and kidney stones [23].

Against this background, we hypothesized that the increased inflammatory potential of dietary intake might also be related to the prevalence of PAD. At present, the role of several common dietary indexes and some food in PAD has been investigated in some studies [24,25]. In 2014, Naqvi et al. reported that the prevalence of PAD was negatively associated with the intake of fiber, folate, and vitamins A, B6, C, and E after adjusting for age, sex, hypertension, diabetes, and smoking [26]. An epidemiological study demonstrated the inverse association between the greater frequency of fruit and vegetable consumption and the risk of PAD [27]. However, in contrast to the DII, which scores 45 nutrients and food components, these studies are limited to a few specific nutrients and fail to provide a comprehensive picture of the relationship between dietary patterns characterized as proinflammatory and anti-inflammatory and PAD. Therefore, our study aimed to assess the impact of DII on PAD based on data from the National Health and Nutrition Examination Survey (NHANES), a population-based cross-sectional survey designed to collect information on the health and nutritional status of adults and children in the United States.

## 2. Materials and Methods

### 2.1. Study Population

Consistent with previous studies investigating PAD using the NHANES database, we analyzed data from the 1999–2004 NHANES cycle (*n* = 31,126) [28]. The website of NHANES provides the details of the study design and protocols (http://www.cdc.gov/nchs/nhanes.htm (accessed on 1 May 2022). Briefly, the NHANES is a survey that uses a complex multistage probability sampling design to estimate the prevalence of major diseases and identify risk factors for diseases. This information will be used to assess the nutritional status of adults and children in the United States to achieve health promotion and disease prevention goals. A total of 7571 participants 40 years or older had a valid ankle–brachial index (ABI) measured. For this study, we excluded participants with a lack of dietary information (*n* = 171), participants with an ABI > 1.4 in at least one leg (*n* = 110, related to incompressible vessels in the leg) [29,30,31], and participants with missing data on covariates of interest (*n* = 1450). Finally, 5840 subjects were included in our study.

### 2.2. Exposure

DII, designed as the exposure variable, is a tool summarized from the literature to assess dietary inflammatory potential via 24-h dietary recall. DII calculates the inflammation effects of dietary consumption from 45 nutrients. The higher the DII score, the greater the proinflammatory effect while the lower the DII score, the greater the anti-inflammatory effect. The method for calculating DII has been reported in detail by N. Shivappa et al. [32]. We must first obtain the Z-score by the following equation: (daily mean intake reported—global daily mean intake)/standard deviation. To minimize the impact of “right skewing”, this value is transformed to a percentile score. Each percentile score is doubled, and then “1” is subtracted to achieve a symmetrical distribution with values centered on 0. The percentile value for each food parameter is then multiplied by its respective “overall inflammatory effect score” to obtain the “food parameter-specific DII score”. Finally, we can achieve an individual “overall DII score” by summing the “food parameter-specific DII score”. In this study, the NHANES 1999–2004 database provides 27 of the 45 food parameters to compute DII. These food parameters and other essential information for the calculation of DII are displayed in Table S1. Previous studies revealed that the DII scores were still available even if the nutrients used to calculate DII were <30 [33,34].

ABI was measured in subjects >40 years old. This measurement method has been reported in the previous studies [26]. In short, systolic blood pressures were measured on the right arm (brachial artery) and both ankles (posterior tibial artery) after a short rest. If the participant’s right arm readings were not available due to any condition that may interfere with accurate measurements (e.g., open wounds, dialysis shunts), the left arm was used for the brachial pressure measurement. Systolic blood pressure was measured twice in subjects aged 40–59 years but only once in subjects aged 60 years and older. The ABI was calculated by dividing the average systolic blood pressure (ASBP) of the ankle by the ASBP in the arm. Since participants aged 60 and older had only one reading, the first reading represented the mean values. PAD was defined as ABI < 0.9 [26,29,35,36].

### 2.3. Covariates

Sociodemographic and lifestyle information was obtained through standardized questionnaires. The Mobile Examination Center (MEC) provided the examination results of the body mass index, blood pressure, and other biochemical parameters. All details of these variables, including the measurement methods, questionnaire data, and variables list, can be found on the official NHANES website (www.cdc.gov/nchs/nhanes/ (accessed on 1 May 2022). Table S2 shows some questions about the sociodemographic and lifestyle information in the questionnaire. We used the Chronic Kidney Disease-Epidemiology Collaboration (CKD-EPI) equation to compute the estimated glomerular filtration rate (eGFR) [37]. CVD history was determined based on self-reported congestive heart failure, coronary heart disease, angina pectoris, heart attack, and stroke. Certified examiners measured the blood pressure using a mercury sphygmomanometer after resting in a seated position for 5 min. The average blood pressure was calculated by the following protocol in NHANES: (1) Diastolic reading with zero is not included in the calculation of the average diastolic blood pressure (ADBP). (2) The ADBP is zero when all diastolic readings are zero. (3) When there is only one blood pressure reading, this reading represents the mean. (4) When there are multiple blood pressure readings, the first reading is not used to calculate the mean value. Hypertension was defined as ASBP/ ADBP ≥ 140/90 mmHg or currently taking antihypertensive medications or previous diagnosis by a doctor or health professional. Diabetes was defined as fasting glucose >7 mmol/L or random glucose ≥11.1 mmol/L or glycated hemoglobin A1c ≥ 6.5% or the usage of hypoglycemic drugs or a history of diabetes. Physical activity was assessed by questions about vigorous activities that resulted in a large increase in breathing or heart rate (e.g., swimming or fast cycling) and moderate activities that resulted in a slight to moderate increase in breathing or heart rate (e.g., golf or recreational cycling). These activities lasted at least 10 min in the past 30 days. We classified physical activity into three levels: less than moderate activity (neither moderate activity nor vigorous activity), moderate activity (no vigorous activity with at least one moderate activity), and vigorous activity (at least one vigorous episode).

### 2.4. Statistical Analysis

Means ± standard deviations were used to represent continuous variables while frequencies or percentages were used to represent categorical variables. Based on the nature of data, we conducted Chi-square, ANOVA, or Kruskal–Wallis H-test to determine differences among participants in different DII quartiles. Three logistic regression models were constructed to assess the association between DII and PAD. Model 1 was an unadjusted model. Age, sex, and race were adjusted in Model 2. Model 3 was adjusted for age, sex, race, BMI, education level, PIR, the level of physical activity, marital status, hypertension, diabetes, CVD, medication use (hypotensive drugs and hypoglycemic drugs), ASBP, ADBP, smoking, TC, HDL-C, eGFR, HbA1c, and CRP. The restricted cubic spline (RCS) was performed to assess the potential non-linear relationship between DII and PAD. We also calculated the Spearman correlation coefficients to evaluate the correlation between DII and some cardiovascular risk factors. Stratification analysis was performed to assess whether the relationships between DII and PAD were affected by age, sex, hypertension, diabetes, CVD, PIR, physical activity, and smoking based on Model 3. A *p* value < 0.05 was considered statistically significant. We used R software (version 4.1, Vienna, Austria) and IBM SPSS statistics version 23.0 (Chicago, IL, USA) to perform all statistical analyses.

## 3. Results

### 3.1. Characteristics of the Study Population

**Figure 1:**
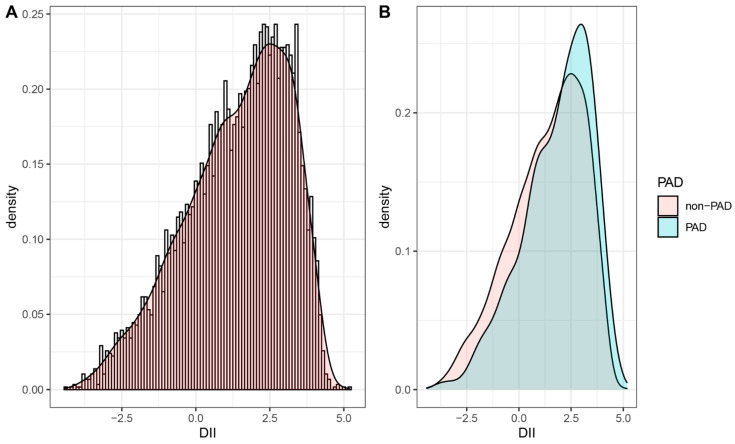
The population distribution of DII. (**A**) Density curve shows the distribution of DII in the total population. (**B**) The distribution of DII in the population with different PAD status. DII, dietary inflammatory index; PAD, peripheral artery disease.

The baseline characteristics of the included participants are shown in Table 1. Among the different quartiles of DII, significant differences were observed in almost all characteristics except diabetes, hypotensive drugs, hypoglycemic drugs, ADBP, HDL, and eGFR. Compared with those with lower DII scores (Quartile 1 and 2), participants with higher DII scores (Quartile 3 and 4) were more likely to be older, female, smokers, and single. In addition, lower physical activity and poorer education levels were more common in participants with higher DII scores. These participants also tended to have higher levels of BMI, ASBP, TC, CRP, and HbA1c while lower levels of PIR (poverty income ratio). More importantly, we observed that participants with a higher DII quartile tended to have higher PAD rates (Quartile 1: 4.79%, Quartile 2: 6.96%, Quartile 3: 7.73%, Quartile 4: 10.33%, *p* < 0.001). Similar results were also observed in the prevalence of CVD and hypertension. Figure 1A illustrates the distribution of DII in the total population, and Figure 1B shows the distribution of DII stratified by PAD status. Participants with PAD preferred a proinflammatory diet.

### 3.2. Association between DII and PAD

Table 2 displays the results of the univariate and multivariable logistic regression analysis. When treating DII as a continuous variable, the increase in DII resulted in a higher prevalence of PAD in the unadjusted model (OR (odds ratios) = 1.190, 95% confidence interval (CI): 1.120–1.265). The association remained statistically significant in Model 2 and 3. When the DII was treated as a categorical variable based on quartiles and using the first quartile as a reference, participants in the third to the fourth quartile had a higher risk of PAD in all three models. The univariate analysis showed that the OR with 95% CI for PAD across increasing quartiles of DII was 1.488 (1.088–2.036), 1.667 (1.226–2.267), and 2.291 (1.708–3.073), respectively. After adjustment for underlying cofounding variables, the OR (95% CI) of PAD throughout the quartiles was 1.231 (0.880–1.722), 1.156 (0.828–1.613), and 1.543 (1.116–2.133), respectively.

**Figure 2:**
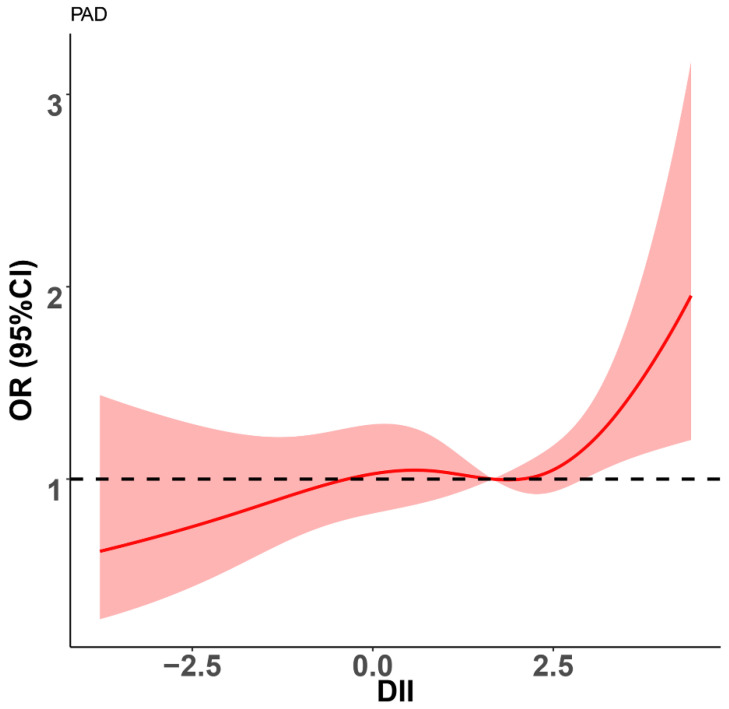
Restricted spline curve shows the relationship between DII and PAD. Red line and red transparent area represent OR and 95% CI, respectively. ORs (95% CI) were adjusted based on Model 3. DII, dietary inflammatory index; PAD, peripheral arterial disease.

As shown in Figure 2, we also used a restricted cubic spline to visualize the association between DII and PAD. In the curve, we found that HR was less than 1 when diet was in anti-inflammatory mode (DII < 0). Thereafter, HR tended to the horizontal line with HR = 1 until DII reached approximately 2.5. Finally, HR sharply increased.

We also performed Spearman correlation analysis to evaluate the correlations between covariates related to CVD and DII. As shown in Table 3, DII was positively correlated with age, ASBP, TC, CRP, and HbA1c (r = 0.035, 0.042, 0.034, 0.140, and 0.083, respectively) while it was negatively correlated with PIR and eGFR (r = −0.167 and −0.027, respectively). However, ADBP and HDL were not significantly correlated with DII (r = −0.013, *p* = 0.333, and r = 0.01, *p* = 0.437).

### 3.3. Stratification Analysis

**Figure 3:**
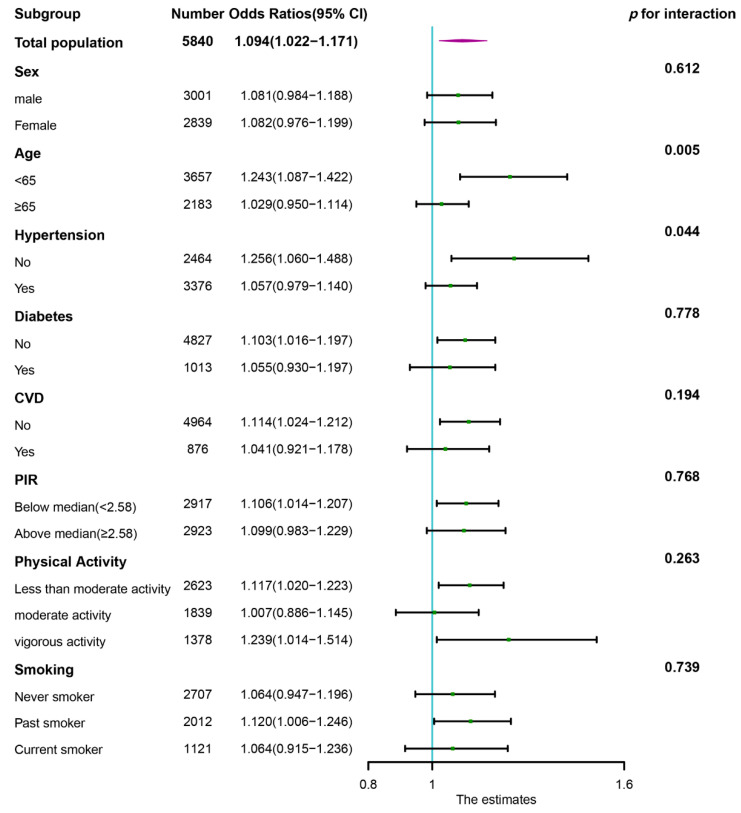
Subgroup analysis of association of DII with PAD. The results were adjusted for all covariates except the corresponding stratification variable. CVD, cardiovascular disease; DII, dietary inflammatory index; PIR, poverty income ratio; PAD, peripheral arterial disease.

Subgroup analysis was conducted according to the categories of age, sex, hypertension, diabetes, CVD, PIR, physical activity, and smoking. The DII was further treated as a continuous variable. As shown in the forest plot (Figure 3), the statistically significant positive associations between DII and PAD were found in participants under 65 years of age (OR = 1.243, 95% CI: 1.087–1.422), participants without hypertension (OR = 1.256, 95% CI: 1.060–1.488), participants without diabetes (OR = 1.103, 95% CI: 1.016–1.197), participants without CVD (OR = 1.114, 95% CI: 1.024–1.212), participants with PIR below the median (OR = 1.106, 95% CI: 1.014–1.207), participants with less than moderate activity (OR = 1.117, 95% CI: 1.020–1.223), participants with vigorous activity (OR = 1.239, 95% CI: 1.014–1.514), and past smokers (OR = 1.120, 95% CI: 1.006–1.246). Furthermore, we also found that the association between DII and PAD was more pronounced in the non-hypertensive population (*p* for interaction = 0.044) and middle-aged population (*p* for interaction = 0.005).

## 4. Discussion

In this large cross-sectional study, a significant positive association between DII and the prevalence of PAD was observed, indicating that a proinflammatory diet might lead to a higher risk of PAD. A restricted cubic spline visualized the relationship between DII and PAD. We observed that the risk of PAD dramatically increased when DII exceeded approximately 2.5. In addition, the stratified analysis revealed a positive association between DII and PAD in population subgroups, which was consistent with the main finding in the total population.

Available evidence suggests that one possible explanation for our results may be the impact of diet on inflammation factors. Ahmad Esmaillzadeh et al. reported that the “Western” diet was positively related to CRP and soluble intercellular adhesion molecule-1 while the healthy diet was inversely related to plasma concentrations of CRP and soluble intercellular adhesion molecule-1 [38]. Likewise, the Dietary Approaches to Stop Hypertension (DASH) diet has been reported to be associated with reduced concentrations of CRP [39]. Recently, more and more studies have demonstrated a positive association between a proinflammatory diet (higher DII scores) and levels of various inflammatory markers: CRP, IL-6, IL-1, IL-2, TNF-α, IFN-γ, and vascular cell adhesion molecule [12,13,40]. Our study also found that the DII was positively associated with CRP (r = 0.140, *p* < 0.001). Meanwhile, previous studies demonstrated that these inflammatory markers (CRP, IL-6, and soluble adhesion molecules) played an essential role in the development of PAD and predicting adverse outcomes in patients with PAD [7,11,41,42]. Therefore, we suspect that a possible mechanism for the prevalence of PAD caused by a proinflammatory diet is an increase in inflammatory markers.

Furthermore, it is well known that dietary nutrient intake plays an important role in the diversity, activity, features, and composition of human gut microbiota [43,44,45,46]. Some studies have demonstrated that the intestinal microbiota can contribute to the development of atherosclerosis through its metabolites [47,48,49]. For example, trimethylamine-N-oxide (TMAO), a pro-atherogenic metabolite formed by gut microbes, has been shown to correlate with PAD severity and prognosis [50,51]. Serum lipopolysaccharide (LPS), another gut derived metabolite, was also elevated in PAD patients and was strongly associated with atherosclerotic burden and oxidative stress [52]. Recently, Eelke Brandsma et al. reported that gut microbiota with proinflammatory characteristics could induce systemic inflammation and accelerate the process of atherosclerosis [53]. Therefore, we should pay attention to the dietary consumption of PAD patients in order to improve their gut microbiota and slow down the progression of PAD.

In this study, we observed that individuals with higher DII scores had a lower family income and poorer education levels, and higher rates of singleness. Meanwhile, growing studies have demonstrated that socioeconomic inequality played an important role in the burden of PAD. Among US adults, the lower the income and education level, the higher the risk of PAD [54,55]. A meta-analysis revealed that unmarried people had a higher risk of CVD and cardiovascular death compared to married people [56]. Moreover, lower physical activity was more common in participants with higher DII scores in our study, which also contributed to a higher risk of PAD. It has been observed that the prevalence of PAD and the incidence of adverse outcomes in patients with PAD are negatively associated with physical activity [28,57]. The intrinsic mechanism can be attributed to the fact that regular exercise reduces visceral fat and induces an anti-inflammatory environment, resulting in an anti-inflammatory effect [58,59]. In addition, Spearman correlation analysis demonstrated that participants with higher DII levels tended to have higher BMI, ASBP, TC, and HbA1c values, which might also increase the burden of PAD in the general population.

The restricted cubic spline visualized the association between DII and PAD. Interestingly, in the curve, we found that the risk of PAD was not increased until DII reached approximately 2.5, which suggests that there might be a threshold to the effect of a proinflammatory dietary pattern on PAD. The level of inflammation developed by the proinflammatory dietary pattern at a DII below 2.5 is not sufficient to cause PAD. On the other hand, the risk of PAD was significantly decreased when diet was in anti-inflammatory mode. Therefore, we need to focus more on people with DII above 2.5 to reduce the burden of PAD and encourage people to eat more foods that are rich in anti-inflammatory nutrients.

Subgroup analyses showed generally consistent associations with the primary results. The positive association between DII and PAD was similar in the population, with differences in age, sex, hypertension status, diabetes status, CVD status, PIR, physical activity, and smoking despite not being statistically significant in some subgroups. However, we found that the ORs in some subgroups, including middle-aged participants and non-hypertensive participants, were significantly higher than those in the corresponding subgroups. We think this may be due to aging and hypertension modifying and attenuating the effect of DII on PAD, which needs to be validated in a larger specific population in the future. Furthermore, we found a positive and statistically significant association between DII and the risk of PAD in participants with vigorous activity. Some studies revealed that excessive exercise and inadequate recovery induced the production and release of proinflammatory cytokines, which might have a detrimental effect on PAD [60,61]. In addition, Ronni E Sahl reported that prolonged excessive exercise could reduce the protective effects of exercise [62]. Therefore, a proinflammatory diet might aggravate systemic inflammation in individuals who participate in vigorous activity, leading to an increased risk of PAD. However, the reduction in sample size after stratification may have led to potential bias, so we need to validate this results in a larger sample size.

Despite the critical findings of our study, some limitations should be mentioned. First, causality cannot be obtained from the cross-sectional study design. Therefore, longitudinal studies with large samples are needed to confirm our results. Second, data on ABI for participants aged <40 years old was not available, which prevented us from analyzing this association for a broad age group. Third, as mentioned in some studies, the information used to calculate DII was collected using a detailed diet questionnaire; however, diet changes over time, so we could not explain the effect of dietary changes on PAD [34]. However, Asghar Z. Naqvi think that 24-h dietary recalls tend to provide highly reliable estimates of recent intakes, and if dietary recalls are collected on all days of the week and all seasons of the year, as is the case with NHANES, then the average of recent intake for a group can yield a reasonable estimate of the average of the usual nutrient intake for that group [26]. Fourth, 24-h dietary recall was used to calculate DII and some covariates were self-reported using validated questionnaires, which can lead to recall bias and social desirability bias. Fifth, physical activity was evaluated by a questionnaire, and actual physical activity could be unclear because of a lack of measurements by a pedometer and so on. Finally, we cannot arbitrarily generalize the results to other populations with different demographics because all participants were US residents.

## 5. Conclusions

In summary, our results demonstrated that a proinflammatory diet was associated with a higher risk of PAD among US adults. Our findings could provide valid information for large-scale prospective studies that could further draw attention to dietary health.

## Figures and Tables

**Table 1:** Baseline characteristics stratified by the dietary inflammatory index (DII) quartiles (Q).

Variables	Total (*n* = 5840)	Q1 (*n* = 1462)	Q2 (*n* = 1465)	Q3 (*n* = 1461)	Q4 (*n* = 1452)	*p*-Value
Age (years)	59.78 ± 12.96	59.16 ± 12.98	59.44 ± 13.03	60.13 ± 12.92	60.4 ± 12.88	0.023
Sex, male, *n* (%)	3001 (51.39%)	948 (64.84%)	794 (54.20%)	680 (46.54%)	579 (39.88%)	<0.001
Hypertension, *n* (%)	3376 (57.81%)	796 (54.45%)	825 (56.31%)	896 (61.33%)	859 (59.16%)	0.001
Diabetes, *n* (%)	1013 (17.35%)	229 (15.66%)	244 (16.66%)	274 (18.75%)	266 (18.32%)	0.096
**MS**, *n* (%)						<0.001
Married/Living with partner	3895 (66.70%)	1067 (72.98%)	1006 (68.67%)	929 (63.59%)	893 (61.50%)	
Widowed/Divorced/Separated	1615 (27.65%)	319 (21.82%)	373 (25.46%)	443 (30.32%)	480 (33.06%)	
Never married	330 (5.65%)	76 (5.20%)	86 (5.87%)	89 (6.09%)	79 (5.44%)	
**Education level**, *n* (%)						<0.001
Less than high school	1875 (32.11%)	360 (24.62%)	437 (29.83%)	498 (34.09%)	580 (39.94%)	
High school diploma or GED	1376 (23.56%)	316 (21.61%)	343 (23.41%)	344 (23.55%)	373 (25.69%)	
More than high school	2589 (44.33%)	786 (53.76%)	685 (46.76%)	619 (42.37%)	499 (34.37%)	
**Physical activity**, *n* (%)						<0.001
Less than moderate	2623 (44.91%)	519 (35.50%)	612 (41.77%)	705 (48.25%)	787 (54.20%)	
Moderate	1839 (31.49%)	484 (33.11%)	497 (33.92%)	439 (30.05%)	419 (28.86%)	
Vigorous	1378 (23.60%)	459 (31.40%)	356 (24.30%)	317 (21.70%)	246 (16.94%)	
**Race**, *n* (%)						<0.001
Mexican American	1205 (20.63%)	307 (21.00%)	299 (20.41%)	283 (19.37%)	316 (21.76%)	
Non-Hispanic white	3261 (55.84%)	873 (59.71%)	851 (58.09%)	806 (55.17%)	731 (50.34%)	
Non-Hispanic black	980 (16.78%)	180 (12.31%)	200 (13.65%)	285 (19.51%)	315 (21.69%)	
Other Hispanic	228 (3.90%)	56 (3.83%)	64 (4.37%)	52 (3.56%)	56 (3.86%)	
Other races	166 (2.84%)	46 (3.15%)	51 (3.48%)	35 (2.40%)	34 (2.34%)	
Hypotensive drugs, *n* (%)	1531 (26.22%)	389 (26.61%)	381 (26.01%)	406 (27.79%0	355 (24.45%)	0.225
Hypoglycemic drugs, *n* (%)	612 (10.48%)	146 (9.99%)	144 (9.83%)	160 (10.95%)	162 (11.16%)	0.553
CVD, *n* (%)	876 (15.00%)	198 (13.54%)	208 (14.20%)	210 (14.37%)	260 (17.91%)	0.004
**Smoking**, *n* (%)						<0.001
Never smoker	2707 (46.35%)	706 (48.29%)	689 (47.03%)	672 (46.00%)	640 (44.08%)	
Past smoker	2012 (34.45%)	570 (38.99%)	498 (33.99%)	495 (33.88%)	449 (30.92%)	
Current smoker	1121 (19.20%)	186 (12.72%)	278 (18.98%)	294 (20.12%)	363 (25.00%)	
PIR	2.80 ± 1.61	3.14 ± 1.61	2.94 ± 1.61	2.68 ± 1.59	2.46 ± 1.56	<0.001
BMI, kg/m^2^	28.43 ± 5.45	28.07 ± 5.23	28.37 ± 5.40	28.66 ± 5.53	28.61 ± 5.62	0.014
ASBP, mmHg	131.04 ± 20.66	129.13 ± 19.21	131.04 ± 20.61	132.04 ± 21.33	131.98 ± 21.33	0.002
ADBP, mmHg	72.91 ± 12.37	73.44 ± 11.68	72.75 ± 12.45	72.62 ± 13.08	72.83 ± 12.23	0.267
Total cholesterol, mg/dL	209.35 ± 41.68	207.68 ± 39.01	207.16 ± 39.98	211.95 ± 44.13	210.62 ± 43.24	0.009
HDL, mmol/L	52.69 ± 16.23	52.52 ± 16.44	52.38 ± 15.72	52.83 ± 16.52	53.03 ± 16.26	0.690
CRP, mg/dL	0.47 ± 0.87	0.38 ± 0.69	0.46 ± 0.74	0.48 ± 1.03	0.58 ± 0.95	<0.001
eGFR, mL/min/1.73 m^2^	83.61 ± 20.02	84.96 ± 18.57	83.59 ± 19.80	83.00 ± 20.91	82.88 ± 20.68	0.131
HbA1c, %	5.77 ± 1.12	5.69 ± 1.04	5.77 ± 1.17	5.80 ± 1.14	5.80 ± 1.11	<0.001
PAD, *n* (%)	435 (7.45%)	70 (4.79%)	102 (6.96%)	113 (7.73%)	150 (10.33%)	<0.001

Values are given as mean ± standard deviation or numbers and percentages. Q1: DII ≤ 0.19; Q2: 0.19–1.66; Q3: 1.66–2.78; Q4: DII > 2.78. ASBP, average systolic blood pressure; ADBP, average diastolic blood pressure; BMI, body mass index; CVD, cardiovascular disease; CRP, C-reactive protein; eGFR, estimated glomerular filtration rate; GED, general educational development; HDL, high-density lipoprotein; HbA1c, glycosylated hemoglobin; LDL, low-density lipoprotein; MS, marital status; PIR, poverty income ratio; PAD, peripheral arterial disease.

**Table 2:** Odds ratios (ORs) and 95% confidence interval (CI) of the DII quartiles for PAD.

DII	Model 1		Model 2		Model 3	
	OR (95% CI)	*p*	OR (95% CI)	*p*	OR (95% CI)	*p*
Continuous	1.190 (1.120–1.265)	<0.001	1.168 (1.095–1.246)	<0.001	1.094 (1.022–1.171)	0.010
Categorical						
Quartile 1 (≤−0.7680)	Reference		Reference		Reference	
Quartile 2 (−0.7680–0.5475)	1.488 (1.088–2.036)	0.013	1.474 (1.066–2.038)	0.019	1.231 (0.880–1.722)	0.224
Quartile 3 (0.5475–1.6080)	1.667 (1.226–2.267)	0.001	1.531 (1.112–2.109)	0.009	1.156 (0.828–1.613)	0.395
Quartile 4 (>1.6080)	2.291 (1.708–3.073)	<0.001	2.106 (1.546–2.869)	<0.001	1.543 (1.116–2.133)	0.009

Data are presented as odds ratios, 95% CIs (confidence intervals), and *p*-value. Model 1 adjusted for none. Model 2 adjusted for age, sex, and race. Model 3 adjusted for all covariates. DII, dietary inflammatory index; PAD, peripheral arterial disease.

**Table 3:** Correlations of DII with some covariates.

	DII	
	r	*p*-Value
Age	0.035	0.007
PIR	−0.167	<0.001
ASBP	0.042	0.002
ADBP	−0.013	0.333
HDL	0.01	0.437
TC	0.034	0.009
CRP	0.140	<0.001
HbA1c	0.083	<0.001
eGFR	−0.027	0.040

ASBP, average systolic blood pressure; ADBP, average diastolic blood pressure; DII, dietary inflammatory index; CRP, C-reactive protein; eGFR, estimated glomerular filtration rate; HDL, high-density lipoprotein; PIR, poverty income ratio; TC, total cholesterol.

## Data Availability

Publicly available datasets were analyzed in this study. This data can be found here: https://wwwn.cdc.gov/nchs/nhanes/ (accessed on 1 May 2022).

## Supplementary Materials

The following supporting information can be downloaded at: https://www.mdpi.com/article/10.3390/nu14173490/s1, Table S1: Food parameters included in our study for the calculation of dietary inflammatory index (DII); Table S2: Some questions about sociodemographic and lifestyle information in the questionnaire.
