# Supplementary material for: A Proinflammatory Diet Is Associated with Higher Risk of Peripheral Artery Disease
Source: Nutrients. 2022 Aug 25;14(17):3490. doi: 10.3390/nu14173490 (PMC9460607; doi:10.3390/nu14173490)
Supplement: Supplementary file 1 [file nutrients-14-03490-s001.zip › Supplementary table S1.pdf]

Table S1. Food parameters included in our study for the calculation of dietary inflammatory index (DII)

Food parameter	Overall inflammatory effect score	Global daily mean intake	Standard Deviation (SD)
Energy (kcal)	0.180	2056	338
Alcohol (g)	-0.278	13.98	3.72
Protein (g)	0.021	79.4	13.9
Fiber (g)	-0.663	18.8	4.9
Total fat (g)	0.298	71.4	19.4
Carbohydrates (g)	0.097	272.2	40.0
Cholesterol (mg)	0.110	279.4	51.2
Omega-3 (g)	-0.436	1.06	1.06
Omega-6 (g)	-0.159	10.80	7.50
Saturated fat (g)	0.373	28.6	8.0
MUFA (g)	-0.009	27.0	6.1
PUFA (g)	-0.337	13.88	3.76
Magnesium (mg)	-0.484	310.1	139.4
Niacin (mg)	-0.246	25.90	11.77
Zinc (mg)	-0.313	9.84	2.19
Fe (mg)	0.032	13.35	3.71
Riboflavin (mg)	-0.068	1.70	0.79
Folic acid (μg)	-0.190	273.0	70.7
Beta-carotene (μg)	-0.584	3718	1720
Caffeine (g)	-0.110	8.05	6.67
Selenium (μg)	-0.191	67.0	25.1
Thiamine (mg)	-0.098	1.70	0.66
Vitamins A (RE)	-0.401	983.9	518.6
Vitamins B_6 (mg)	-0.365	1.47	0.74
Vitamins B_{12} (μg)	0.106	5.15	2.70
Vitamins C (mg)	-0.424	118.2	43.46
Vitamins E (mg)	-0.419	8.73	1.49

MUFA, monounsaturated fatty acids; PUFA, polyunsaturated fatty acids.

All food parameters were used as continuous type variables to calculate the DII.
