# Supplementary material for: A Proinflammatory Diet Is Associated with Higher Risk of Peripheral Artery Disease
Source: Nutrients. 2022 Aug 25;14(17):3490. doi: 10.3390/nu14173490 (PMC9460607; doi:10.3390/nu14173490)
Supplement: Supplementary file 1 [file nutrients-14-03490-s001.zip › Supplementary table S2.pdf]

Table S2. Some questions about sociodemographic and lifestyle information in the questionnaire

Variable	questionnaire data
Education	(Sample Person (SP) Interview Version) What is the highest grade or level of school {you have/SP has} completed or the highest degree {you have/s/he has} received?
Physical activity	1. The next questions are about physical activities including exercise, sports, and physically active hobbies that {you/SP} may have done in {your/his/her} leisure time or at school over the past 30 days. First I will ask you about vigorous activities that cause heavy sweating or large increases in breathing or heart rate. Then I will ask you about moderate activities that cause only light sweating or a slight to moderate increase in breathing or heart rate. Over the past 30 days, did {you/SP} do any vigorous activities for at least 10 minutes that caused heavy sweating, or large increases in breathing or heart rate? Some examples are running, lap swimming, aerobics classes or fast bicycling.<br>2. [Over the past 30 days], did {you/SP} do moderate activities for at least 10 minutes that cause only light sweating or a slight to moderate increase in breathing or heart rate? Some examples are brisk walking, bicycling for pleasure, golf, and dancing.
Smoking	1. These next questions are about cigarette smoking and other tobacco use. {Have you/Has SP} smoked at least 100 cigarettes in {your/his/her} entire life?<br>2. {Do you/Does SP} now smoke cigarettes.
Hypertension	1. {Have you/Has SP} ever been told by a doctor or other health professional that {you/s/he} had hypertension, also called high blood pressure?<br>2. In the past month, {have you/has SP} used or taken medication for which a prescription is needed? Do not include prescription vitamins or minerals you may have already told me about. (Prescription drug categories are determined based on U.S. Food and Drug Administration National Drug Code Directory. The website is https://wwwn.cdc.gov/Nchs/Nhanes/1999-2000/RXQ_RX.htm#FDACODE1. The code of hypotensive drugs is 506.)
Diabetes	1. The next questions are about specific medical conditions. {Other than during pregnancy, {have you/has SP}/{Have you/Has SP}} ever been told by a doctor or health professional that {you have/{he/she/SP} has} diabetes or sugar diabetes?<br>2. In the past month, {have you/has SP} used or taken medication for which a prescription is needed? Do not include prescription vitamins or minerals you may have already told me about. (Prescription drug categories are determined based on U.S. Food and Drug Administration National Drug Code Directory. The website is https://wwwn.cdc.gov/Nchs/Nhanes/1999-2000/RXQ_RX.htm#FDACODE1. The code of hypoglycemic drugs is 1036.)
Cardiovascular disease	1. Has a doctor or other health professional ever told {you/SP} that {you/s/he} . . .had congestive heart failure?<br>2. Has a doctor or other health professional ever told {you/SP} that {you/s/he} . . .had coronary heart disease?<br>3. Has a doctor or other health professional ever told {you/SP} that

	{you/s/he} . . .had angina, also called angina pectoris?<br>4. Has a doctor or other health professional ever told {you/SP} that {you/s/he} . . .had a heart attack (also called myocardial infarction)?<br>5. Has a doctor or other health professional ever told {you/SP} that {you/s/he} . . .had a stroke?
hypotensive drugs and hypoglycemic drugs	In the past month, {have you/has SP} used or taken medication for which a prescription is needed? Do not include prescription vitamins or minerals you may have already told me about. (Prescription drug categories are determined based on U.S. Food and Drug Administration National Drug Code Directory. The website is https://wwwn.cdc.gov/Nchs/Nhanes/1999-2000/RXQ_RX.htm#FDACODE1. The code of hypoglycemic drugs is 1036, and the code of hypotensive drugs is 506.)
